# Potentially Toxic Elements Uptake and Distribution in *Betula middendorffii T.* and *Duschekia fruticosa R.* Growing on Diamond Mining Area (Yakutia, Russia)

**DOI:** 10.3390/plants13172440

**Published:** 2024-08-31

**Authors:** Anna Gololobova, Yana Legostaeva

**Affiliations:** Diamond and Precious Metal Geology Institute, Siberian Branch of the Russian Academy of Sciences, Yakutsk 67700, Russia

**Keywords:** potentially toxic elements (PTEs), bioaccumulation factor (*BAF*), contamination, accumulation, mining area

## Abstract

This study was conducted in the territory of the industrial site of the Udachny Mining and Processing Division (Yakutia, Russia). The objects of study were permafrost soils and two species of shrubs (*Betula middendorffii T.* and *Duschekia fruticose R.*). Soil and plant samples were analyzed by atomic absorption spectrometry for the presence of potentially toxic elements (Pb, Ni, Mn, Cd, Co, Co, Cr, Zn, Cu, and As). The bioaccumulation factor for each element was also calculated. In the studied plants, the investigated elements were arranged in the following descending row in terms of their content: Mn > Zn > Cr > Ni > Cu > Pb > As > Co > Cd, but in terms of bioaccumulation degree, they decrease in the following row: Cr > Zn > Ni > Mn > Pb > Cu > Cd > Co—for *Betula middendorffii*, Cr > Zn > Ni > Pb > Cu > Mn > Mn > Cd > Co—for *Duschekia fruticose*. The bioaccumulation factor results confirmed that *Betula middendorffiii* and *Duschekia fruticosa* are resistant to high concentrations of Cr, Ni, Co, Cu, Mn, and Zn elements coherent to kimberlites.

## 1. Introduction

Of all anthropogenic activities, mining activities are considered to contribute significantly to environmental pollution [1,2]. In the mining industry, open pit mining is the most efficient, but the dispersion of ore elements results in the formation of polyelemental anthropogenic anomalies covering all components of the biosphere [3]. In the process of mineral processing, solid particles containing potentially toxic elements (PTEs) are released [4]. The amount of PTEs in the environment has increased due to mining activities over the last decades [5]. PTEs come predominantly from waste disposal facilities, including tailings ponds, waste rock dumps, sludge dumps, etc. [6]. Ultimately, dust dispersion contributes to the release of PTEs from mining activities into the environment, primarily contaminating soils [7,8]. The contamination of soils with PTEs leads to their uptake and accumulation by plants [9,10] and thus has a toxic effect, causing degradation of plant communities [11]. Plants growing on contaminated soils are more likely to accumulate potentially toxic elements than plants growing on unpolluted soil [12,13].

The study of the accumulation of PTEs from soil into plants to assess soil ecotoxicity is of particular interest [10], since plants can remove, decompose, or neutralize pollutants [14], thereby cleaning up contaminated soil. Thus, the study of the bioavailability of PTEs is important for understanding the persistent anthropogenic load in these areas.

Currently, there are practically no studies on the bioaccumulation of PTEs in plants growing in the territory of this type of industrial area, namely diamond mines. Therefore, the purpose of this paper is to determine the concentrations of PTEs in plants and to assess their ability to accumulate the PTEs. This study can provide data on the resistance of local plants to high concentrations of PTEs, as well as provide insight into their behavior in contaminated environments for monitoring.

## 2. Materials and Methods

### 2.1. Research Area

**Figure 1:**
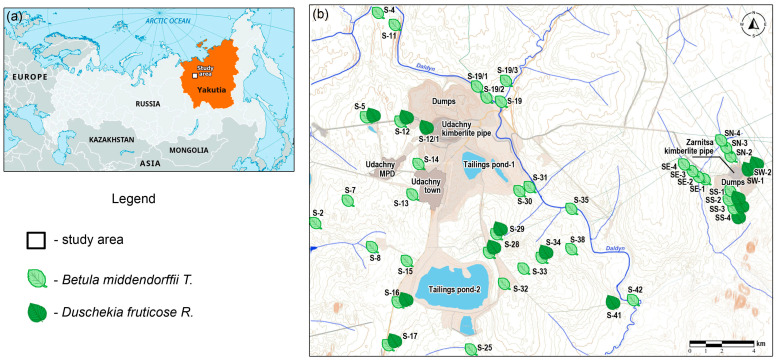
Location of the study area: (**a**) location of the study area; (**b**) *Betula middendorffii T.* and *Duschekia fruticose R.* sampling scheme.

Biogeochemical studies were conducted in the territory of the Daldyn kimberlite field, located in the central part of the Daldyn–Alakit mining and industrial district in the northwest of Yakutia. Within the Daldyn kimberlite field there is an industrial site of the Udachny mining and processing division, where two primary diamond deposits are being developed: the Udachny and the Zarnitsa kimberlite pipes (Figure 1).

The territory of the Daldyn–Alakit area is located in the zone of continuous spreading and close occurrence of permafrost. The relief of the trappean plateau is hilly semi-gently and semi-steeply sloping with the absolute mark of 400–500 m and relative elevations above the nearest watercourses of 100–250 m [15]. The climate is sharply continental. The mean annual temperature is 12.7 °C, and the amplitude of maximum and minimum mean monthly data ranges from −41.6 to 14.8 °C. The average annual precipitation is 200–250 mm, and 75–80% of it occurs during the warm season [16].

### 2.2. Sample Collection and Preparation

Considering the source of trace element emissions in the study area, plant samples were collected in the vicinity of the industrial zone of the Udachny mining and processing division (near kimberlite pipe open pits, dumps, tailings ponds, the processing plant, and other anthropogenic facilities).

It is widely known that plant leaves are one of the most informative indicators of the environmental condition [17,18]. At the initial stages of the search for primary diamond deposits in the study area, the geobotanical studies revealed that larch-sparse forests with alder were fragmentarily distributed, associated with the outcrops of the kimberlite pipes and partially with their dealluvial plumes. The studies also show that the stand type changes much less than the sinusial structure of the ground cover—moss/lichen and shrub layer. Thus, the indicative role of the shrub layer, for example, is much higher. Therefore, it is more effective to carry out the bioindication of the state in the soil–vegetation system by biogeochemical response on the example of leaves of shrubby plants, acting not only as indicators of plicate structures but also of changes in the general ecological–geochemical state of the substrate [16]. The plants selected for this study were representatives of the most common species of shrubs in the northern taiga: *Betula middendorffi T.* and *Duschekia fruticose R*., and the objects of study were leaves.

The leaf plate is a powerful air pump of plants and contributes to the absorption and accumulation of pollutants entering with industrial emissions [19]. The leaves of dominant shrub species were sampled using the mid-sample method at a height of 1.0–1.1 m from the soil surface on the outer side of the crown in a circular pattern. For analysis, the leaves were taken without petioles. The selected leaves were carefully cleaned of dust and packed into numbered paper bags. Samples were stored in well-ventilated containers during transportation. The samples of plants were prepared for chemical and analytical tests according to generally accepted methods [20].

To calculate the bioaccumulation, soil samples were collected at the same observation points as the plants, describing the main morphological characteristics. All observation points were recorded using a global positioning system (GPS) device. Soil samples were collected from the surface layer using a stainless steel shovel to avoid heavy metal contamination of the soil. The soil samples were then air-dried in the laboratory at room temperature, disaggregated with a pestle and mortar, and sieved through a 2 mm sieve mesh [21]. All soil samples were gathered very carefully and transported to the laboratory for analysis.

Analysis of mobile forms of PTE in soils and plants was carried out by digesting 1 g of sample in 10 mL of 1 N HNO_3_. 1 N HNO_3_ was used as an oxidizing agent due to its high efficiency in digesting carbonates, phosphates, and other components to determine the content of mobile forms of elements in the samples, in contrast to 1 N HCl [22,23,24]. The PTEs in plants and soils were determined using an atomic absorption spectrophotometer (AAS) model MGA-1000 (GC Lumex, Saint Petersburg, Russia) after acid decomposition and extraction with 1N HNO_3_ according to the methodology M 03-07-2014 [25].

Furthermore, the soils were analyzed for pH and organic matter content. The pH in soil/water suspension 1:2.5 was measured using a glass electrode according to ISO 10390:2005 [26], GOST-26483-85 [27]. The content of the soil total organic matter (SOM) was determined by sulfochrome oxidation according to ISO 14235:1998 [28], GOST-26213-91 [29]. Total nitrogen (TN) was calculated using the spectrophotometric method (PE-5300VI Russia) according to state standards [30,31]. The granulometric composition of the soil was determined by sedimentation analysis using the pipette method [23].

All analyses were performed in triplicate relative to a control for analytical accuracy. The quality of the analysis was ensured by the analysis of certified standard soil samples SDPC-1 (Albic Podzols) and SSC-1 (Haplic Calcisols) and birch leaf LB-1 (GSO 8923-2007). The observational error was less than 0.5% at *p* = 0.95. 

### 2.3. Determination of Bioaccumulation Factor (BAF)

The bioaccumulation factor gauges the concentration of contaminants gathered by plants from soil, revealing their uptake efficiency [32]. The *BAF* was calculated using the following equation [33]:

$$BAF=\frac{C_{p\;}}{C_{s}}$$

where *C_p_* and *C_s_* are the concentrations of the element in plants and soil samples, respectively. The plants with *BAF* values above 1 demonstrate the potential success of the plant species for phytoremediation purposes [34]. The *BAF* is not a constant, and even in the same species it can vary 100–1000 times during its lifetime. When the *BAF* is >1, phytoaccumulation occurs, and when *BAF* is ≤1, there is no phytoaccumulation, i.e., the element content in ash is lower than in soil [35]. Additionally, an additional ranking was introduced by analogy with the coefficient of biological accumulation and capture proposed by B.B. Polynov (1948) and further developed by A.I. Perelman (1989) [36]: *BAF* = 0–1—the group of weak capture of the element, no accumulation; *BAF* = 1–10—weak accumulation and medium capture; *BAF* = 10–100—the vigorous accumulation; *BAF* ≥ 100—the serious (high) accumulation [37].

### 2.4. Statistical Analysis

Before the statistical analysis, the distribution of the dataset was assessed using the Kolmogorov–Smirnov (*p* < 0.2) and Shapiro–Wilk (*p* < 0.05) methods; if the distribution value was not normal, the data were transformed according to the principles of compositional data analysis (CoDa) [38,39,40] using centered logarithmic ratio (clr) conversion.

All statistical analyses were processed using Statistica v. 13.0, IBM SPSS Statistics v. 23.0 software, and OriginPro 2024.

## 3. Results and Discussion

### 3.1. Concentrations of PTEs in Soils and Plants

The soils of the Daldyn–Alakit field are classified as Oxyaquic Turbic Cryosols (Eutric and Loamic). The average temperature for three summer months (June–August) varies from 0.1 to 3.6–3.9 °C at a depth of 20 cm for zonal loamy soils of watersheds [41]. The lower horizons of soils (C or CRM) are usually in a frozen state even in the summer months. The content of hygroscopic moisture ranges from 0.98% to 3.15% depending on the depth of the horizons and the content of coarse-humus organic matter.

The acid-base conditions of the soil vary from strongly acidic to alkaline (pH = 4.3–8.7) (Table 1). The proportion of weak- and medium-acidic pH is 15%, the proportion of neutral is 15%, and alkaline is 70%, which characterizes the most of soil samples. The content of soil organic matter was quite high.

The concentrations of PTEs determined in soils and plant leaves are presented in Table 2. The PTEs mean concentrations (mg/kg) in soils are arranged in the following order: Mn > Ni > Zn > Cu > Co > Co > Pb > Cr > As > Cd. The contents of some elements, such as nickel, chromium, cobalt, arsenic, and manganese, exceeded background values by 4.93, 2.09, 1.72, 1.69, and 1.65 times, respectively. The PTEs content variations in soils were assessed using the coefficient of variation (CV), which indicates the degree of variability in relation to the sample mean values. When the coefficient is higher than 35%, the variation level is strong; at 35–15%, it is medium, and if the coefficient is lower than 15%, the variation is considered to be weak [42,43]. In the studied area, there is a high variability in the concentration values of Mn, Ni, Zn, Co, and Cu; medium for Cr and low for Pb, Cd, and As.

The orders of distribution of mean concentrations of PTEs in both plant species were similar and located in the following descending order: Mn > Zn > Cr > Ni > Cu > Pb > As > Co > Cd, except for Zn, which was below the detection limit in *Duschekia fruticosa* leaves. In *Betula middendorffii* leaves, the concentrations of nickel, manganese, cadmium, chromium, and zinc exceeded the values in *Duschekia fruticosa* leaves by 1.2, 4.6, 1.4, 2.7, and 1.0 times, respectively. The coefficients of variation were high for Mn in both leaf species and for Zn for *Betula middendorffii* leaves.

Soil phytotoxicity is the soil property to suppress the growth and the development of vascular plants. The necessity to determine this parameter appears when monitoring chemically contaminated soils [37]. The analysis of the average content of PTEs in soils relative to the total concentrations in the surface layer of soils, which are considered limiting with respect to phytotoxicity, as first introduced in the works of A. Kabata Pendias and H. Pendias [44], divided the elements into three groups (Table 3):

I—Pb and As with KF < 0.1;

II—Cd, Cr, Zn, and Cu with 0.1 < KF < 1.0;

III—Ni, Mn, and Co with KF > 1.0.

The first group includes Pb and As in the soils of the territory of the Daldyn kimberlite field in concentrations that are not toxic to plants. The second group of elements also does not exceed the limits of phytotoxicity and characterizes the influence of kimberlite (Cr and Cu) and dolerite (Zn) components in soils. The third group of elements is characterized by Ni, Mn, and Co, both in soils of background areas and at the industrial site. The subsoils and the soils in the zone of impact of quarries and tailing pits are generally depleted in available forms of all analyzed elements. This is probably the reason that despite the 42-year operation period of the quarry on its sides and in the zone of its impact with a strip width of 700–800 m, there is no vegetation regeneration except for single pioneer ruderal species.

According to calculations in Table 3, it should be noted that in the leaves of *Betula middendorffii* and *Duschekia fruticosa,* Ni and Cr are in excessive concentrations for plants, which is a consequence of kimberlite magmatism in soils and plants.

### 3.2. Bioaccumulation Factor (BAF)

Northern taiga forests in the Daldyn river basin consist of almost the only tree species, *Larix gmelinii*. The undergrowth in larch redwoods or purely shrub thickets is formed by *Betula middendorffii*, *Duschekia fruticosa*, and various species of willows. The density of the stand is 0.3 and higher, so the role of the shrub layer in northern taiga landscapes is very high. The most widespread shrubs of *Betula middendorffii* and *Duschekia fruticosa* were chosen as BAF indication objects. And to be able to interpret the process of bioconcentration in the soil–plant system, the *BAF* was calculated for each survey site. The values of plant bioaccumulation factors in the survey area are presented in Table 4.

The average *BAF* values for birch were arranged in the following order: Cr > Zn > Ni > Mn > Pb > Cu > Cd > Co, for alder: Cr > Zn > Ni > Pb > Cu > Mn > Cd > Co. The only difference was the lower Mn content in alder leaves. Regardless of the plant species, Cr had the highest phytosorption potential, followed by Zn and Ni. The proportion of absorbed elements with *BAF* > 1 in the studied plant species was for zinc—97%, chromium—94%, nickel—86%, lead—75%, copper—39%, manganese—8% in *Betula middendorffii* leaves; zinc—100%, chromium—100%, chromium—100%, nickel—93%, lead—64%, and copper—43% in *Duschekia fruticosa* leaves.

The absence of Cd and Co accumulation in birch leaves and Cd, Co, and Mn accumulation in alder leaves was noted. The highest phyto-absorption in birch leaves was observed in points S-11 for Pb, Mn, Cr, and Cu, S-32 for Ni, and S-15 for Zn. In alder leaves, the maximum photoabsorption was found in the studied points S-17 for Pb, S-29 for Ni, S-12 for Cr, S-16 for Zn, and SS-4 for Cu. It was established that the greatest amount of trace elements is accumulated by plants in the observation point S-11, which is located at the exhausted quarry for extraction of construction materials for road filling, where on the surface are formed on basitic rocks.

The occurrences of kimberlite magmatism in the study area are displayed in the geochemical field on the regional ancient and modern landscape background in the form of specific primary and secondary biogeochemical halos of a wide range of elements, representing a kind of natural geochemical anomaly. The main indicator elements are Cr, Ni, Co, Zr, Li, Cu, Ba, Nb, Ti, Sr, Ga, Sc, V, and Mn [48,49,50,51]. Cr and Ni are typomorphic for diamondiferous varieties of kimberlites. Accordingly, the accumulation of these elements in soils and vegetation in the territory of kimberlite fields is quite natural, thus explaining the geochemical inheritance of Cr, Ni, Mn, and Cu by both *Betula middendorffii* and *Duschekia fruticosa*.

**Figure 2:**
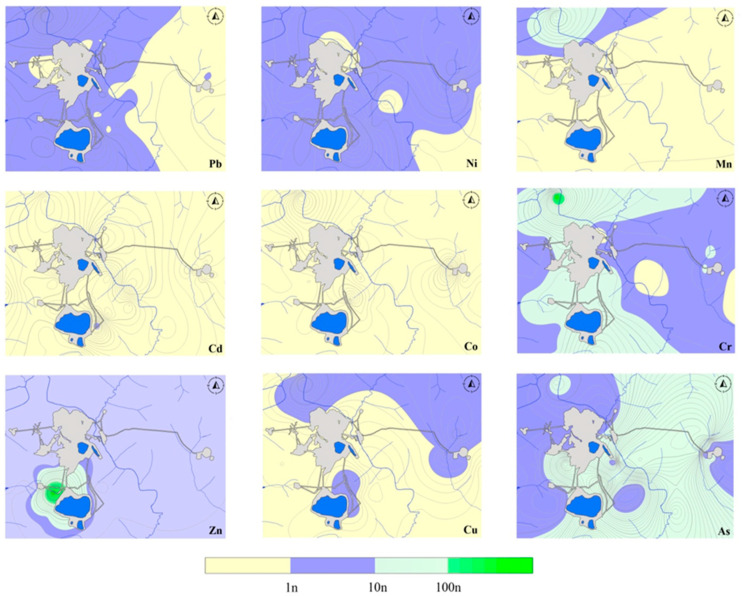
Bioaccumulation factor in leaves of *Betula middendorffii. BAF* = 0–1—no accumulation, the weak capture; *BAF* = 1–10—weak accumulation and medium capture; *BAF* = 10–100—the vigorous accumulation; *BAF* ≥ 100—the serious (high) accumulation.

The analysis of the spatial distribution of BAF for the two shrub species showed different results. In northern taiga landscapes, *Betula middendorffii* is distributed ubiquitously on terrigenous carbonate formations, horizons of spotted, banded, and calcitized dolomites, dolerites, tuffs, tuffobreccias, and tuffogenic formations. It was proved in early studies [16] that *Betula middendorffii* is able to accumulate more trace elements and has a greater potential for phytoextraction. The highest BAF values are characterized by areas in the zone of impact of mining facilities—quarries and waste rock dumps of the Udachny and Zarnitsa kimberlite pipes, as well as enrichment—tailing pits, underground injection sites of highly mineralized brines (Figure 2). *Betula middendorffii* is characterized by high resistance to the content of trace elements in soils while maintaining homeostasis of ontogenesis.

In general, the spatial analysis of BAF confirmed the conclusions about the absence of Cd, Co, and Mn bioaccumulation in birch leaves, both in the areas of ore-bearing rock formation and on the periphery of the Udachny and the Zarnitsa kimberlite pipes. In addition, BAF values for these elements do not increase under technogenic transformation, despite the increase in the concentration of Cd, Co, and Mn in technosols. The phytoaccumulation of Cr, As, and Zn can be explained by the increase in the concentration of these elements within the boundaries of a technogenic anomaly in the soils of the industrial site of the mining and processing plant.

In recent years, there has been significant progress in deciphering the molecular mechanisms of the perception of various compounds [52]. It is known that trace elements affect many aspects of the cell metabolism. Cationic forms of PTE can associate with carboxyl, hydroxyl, phosphate, and amine groups, thereby inducing changes in the elements that comprise these groups, ranging from nucleotides, proteins, coenzymes, and phospholipids. The suppression of enzyme systems results in impaired respiration processes and protein synthesis and compromised cytoplasmic membrane functions [53,54]. For example, chromates absorbed by plant roots affect the identification of regulatory proteins that control multilevel dynamics of the oxyanion. Cr (VI) reduces the expression of several glutamate receptors, whereas amino acids, such as glutamate, cysteine, and proline, provide plant protection from the stress induced by hexavalent chromium and enable the tissues to balance growth and protection in the conditions of oxidative damage produced by Cr (VI) [52]. As a result of plant root absorption, Cu is transported acropetally along the xylem and is present in the exudate of the xylem of the negatively charged complexes, possibly including amino acids (AAs) [55]. Transported to the leaves, metals accumulate in the vacuoles of the epidermis [56,57] and its derivatives, i.e., trichomes [58] and glands [59], both of which function to remove metals from the plant body. The developed mechanism may prove valuable in explaining the mechanisms of a better adaptation of plants to biotic and abiotic challenges. The highly contrasting plant responses exhibited at the transcriptional and translational levels are dependent on elemental concentrations in the soil and fit well with the concept of hormesis, an adaptive mechanism that provides plant resistance to environmental challenges, including high elemental concentrations in soils caused by telescoping manifestations of kimberlite anomalies. The manifestations of adaptive mechanisms include tolerance of birch to high concentrations of chromium, arsenic, and zinc.

**Figure 3:**
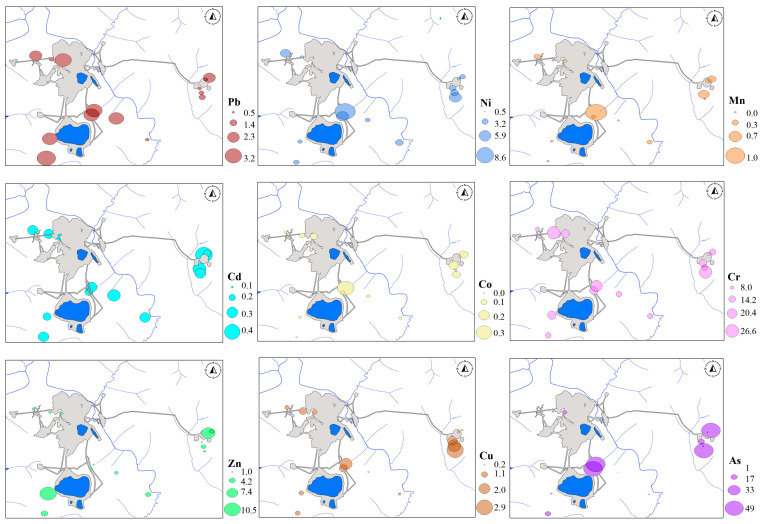
Bioaccumulation factor in leaves of *Duschekia fruticose. BAF* = 0–1—no accumulation, the weak capture; *BAF* = 1–10—weak accumulation and medium capture; *BAF* = 10–100—the vigorous accumulation; *BAF* ≥ 100—the serious (high) accumulation.

The larch forests with *Duschekia fruticosa* are formed in patches in the upper part of slopes and are confined to kimberlite outcrops and partially to their deluvial plumes. Kimberlite bodies break through the dolomite horizon, and in the places of their outcrops, more favorable conditions of moisture and drainage are created than in the host carbonate sediments. The larch forests with *Duschekia fruticosa* associated with kimberlite bodies stand out sharply against the background of larch lichen sparse forests related to carbonate rocks. This is also reflected in the elemental composition of the soils, with the predominance of kimberlite-coherent associations. The Udachny pipe quarry has been mined since 1982, and the Zarnitsa pipe has been developed since 1998. The main forest areas with *Duschekia fruticosa* are already buried under anthropogenic landscapes. The fragmentally preserved areas fringe the periphery of the kimberlite field (Figure 3). BAF values are highly variable, with maxima confined to impact zones of anthropogenic impact.

In contrast to *Betula middendorffii,* the leaves of *Duschekia fruticose* do not indicate phytoporption of Mn, Cd, and Co, despite their high concentrations in soils. High BAF values are characterized by areas near quarries and tailing dumps with high phytop absorption of elements coherent with Cr and Ni kimberlites.

Rapid accumulation of elements, Ni, Cr, and Cu in particular, has been attributed to the high content of low molecular weight acids such as malic and malonic acids in plants that grow on soils formed on acidic or ultrabasic rocks. Kimberlites and dolerites of the Daldyn kimberlite field are precisely magmatic ultrabasic rocks of extrusive facies. Consequently, the maximum accumulation of Ni, Cr, and Cu in the leaves of *Duschekia fruticose* is entirely natural.

Unstable plant species have a limited ability to transfer metal ions into the tonoplast, so they accumulate such elements, including PTEs, in the form of slow-moving compounds in the cytoplasm directly. It appears that in deciduous shrubs, a significant portion of PTEs accumulates in the chloroplast, which ensures the stability of ontogeny under natural conditions of high concentrations of a large number of trace elements in soils and rocks. This is exactly what we observe in the example of *Duschekia fruticose*. Whereas *Betula middendorffii* is tolerant to soil and rock variability and therefore is widespread in northern taiga landscapes, however, it shows the strongest response to the effects of excessive PTE concentrations.

**Figure 4:**
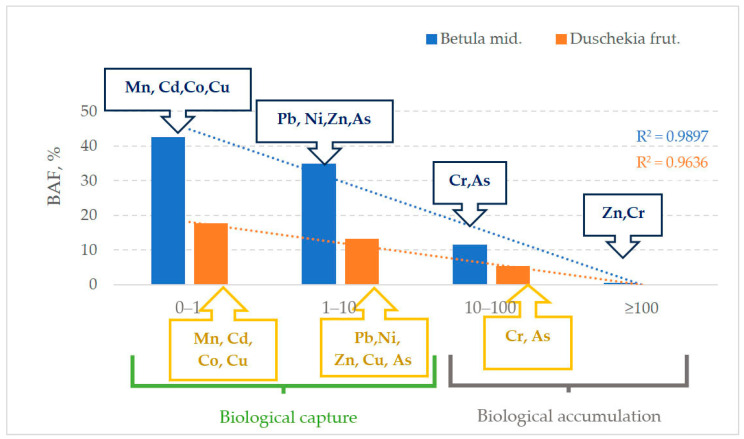
The level of biological capture and accumulation of PTEs in the soil–plant system on the territory of the industrial site of the Udachny Mining and Processing Division.

The analysis of the values of BAF at each point of observation generally confirms the identified regularities (Figure 4).

Thus, in the leaves of *Betula middendorffii* and *Duschekia fruticose*, the absence of accumulation and weak biological capture are characterized by Mn, Cd, Co, and partially Cu. At the level of weak accumulation, the two studied species differ in variations of Ni and Cu accumulation. The vigorous accumulation occurs in 5.4 and 11.6 percent for chromium and arsenic in the leaves of *Duschekia fruticose* and *Betula middendorffii*, respectively. In addition, strong accumulation of Zn and Cr in the leaves of *Betula middendorffii* was recorded at the two points located near the tailings dump.

Summarizing the conducted research, it should be noted that the geochemical situation in the soil–plant system that has developed in the territory is a consequence of natural and anthropogenic conditions that distinguish the characteristics of the rocks of the Daldyn kimberlite field and the consequences of the stages of development of the diamondiferous deposits.

## 4. Conclusions

The response of a biological system to geochemical stress is a generalized system that activates the expression of a number of genes and ensures the survival of the cell under extreme conditions. At the same time, many intracellular detoxification mechanisms and processes limiting entry of PTEs into plants are non-selective.

The indicator role of the shrub layer is clearly shown when analyzing the geological structure and relief of the territory. The constructed schemes of spatial bioindication display the stability of *Betula middendorffii* and *Duschekia fruticose* ontogenesis to high variations in the content of trace elements in soils, which are a consequence of their natural and anthropogenic anomalies, as a result of the combined impact of the geological environment and anthropogenic factor.

Generally, the analysis of bioaccumulation coefficient in the soil–plant system in the territory of mining and processing plants has revealed that shrubs *Betula middendorffii T.* and *Duschekia fruticosa R.* show resistance to high concentrations of elements coherent to kimberlites—Cr, Ni, Co, Cu, Mn, Zn.

There are a number of mechanisms in plants that provide detoxification of the PTE ions and resistance to the stress induced by them. The data on physiological and biochemical responses of organisms, including higher vascular plants, are very contradictory and insufficient. There are very few studies of plant physiology and their development in the areas of biogeochemical provinces associated with the manifestation of rock magmatism and tectonic disturbances, and they are concentrated primarily in agro-territories. Nevertheless, these types of studies are important for understanding the mechanisms involved in the detoxification of PTEs and for understanding the processes of bioaccumulation in relation to different concentrations of PTEs.

## Figures and Tables

**Table 1:** Physicochemical characteristics of soils in the study area.

Index		Mean	Geometric Mean	Median	Minimum	Maximum	Variance	Std. Dev.
Physicochemical properties	pH	7.62	7.56	7.80	4.32	9.30	0.72	0.85
	Humus, %	9.88	7.01	6.20	1.10	47.00	72.52	8.52
	SOC, %	5.73	4.07	3.60	0.64	27.26	24.40	4.94
	TN, %	0.81	0.62	0.76	0.03	1.98	0.23	0.48
	SOC/TN	7.06	6.56	4.72	25.52	13.80	106.55	10.32
Granulometric fractions	1–0.25 mm	1.04	0.44	0.34	0.05	2.81	1.47	1.21
	0.25–0.05 mm	7.65	6.66	6.26	3.00	12.19	18.06	4.25
	0.05–0.01 mm	23.45	22.57	26.62	15.61	30.86	47.76	6.91
	0.01–0.005 mm	10.34	10.31	10.19	9.55	11.55	0.68	0.83
	0.005–0.001 mm	15.83	15.28	15.72	11.67	23.66	24.01	4.90
	<0.001 mm	25.21	24.12	27.92	12.70	29.74	49.90	7.06
	<0.01 mm	51.38	51.24	50.02	46.55	57.71	18.09	4.25
	>0.01 mm	32.14	31.43	33.08	22.14	42.59	55.80	7.47

**Table 2:** The PTE content in soils and leaves of plants in the study area, mg/kg.

Index		Pb	Ni	Mn	Cd	Co	Cr	Zn	Cu	As
Soil	Mean	2.21	15.39	311.63	0.14	4.53	1.94	12.10	8.21	0.22
	Geom. mean	1.80	3.43	199.9	0.106	2.73	0.967	9.36	6.14	0.12
	Minimum	0.33	1.00	34.76	0.03	0.53	0.11	0.05	0.82	0.03
	Maximum	6.60	163.8	1856	1.12	34.65	21.45	36.46	28.44	0.84
	Standard deviation	1.41	40.97	415.5	0.18	6.92	4.08	6.99	6.40	0.21
	Variance	1.99	1678	172,608	0.03	47.94	16.62	48.91	40.95	0.04
	Background value	1.79	3.12	189	0.11	2.64	0.93	9.47	5.81	0.13
*Betula middendorffii*	Mean	3.03	8.08	144.8	0.04	0.39	12.30	42.94	4.73	ND
	Geom. mean	2.85	7.70	55.64	0.03	0.27	12.19	37.48	4.59	ND
	Minimum	1.51	4.00	14.67	0.03	0.13	9.92	25.00	2.92	ND
	Maximum	7.33	15.9	2237	0.26	2.71	15.62	97.54	8.83	ND
	Standard deviation	1.15	2.61	408.5	0.04	0.46	1.67	24.18	1.27	ND
	Variance	1.33	6.81	166,887	0.002	0.22	2.79	584.6	1.62	ND
*Duschekia fruticosa*	Mean	3.09	6.81	31.5	0.03	0.15	12.08	ND	5.16	ND
	Geom.mean	2.98	6.63	25.71	0.03	0.14	11.98	ND	5.08	ND
	Minimum	2.14	3.45	9.65	0.03	0.13	9.60	ND	4.13	ND
	Maximum	4.58	9.02	98.9	0.05	0.44	14.49	ND	7.04	ND
	Standard deviation	0.86	1.47	24.2	0.01	0.08	1.59	ND	0.97	ND
	Variance	0.74	2.153	586.1	0.0001	0.007	2.54	ND	0.936	ND
	Background values	2.88	7.39	44.82	0.03	0.22	12.13	33.46	4.72	ND

Note: ND—below the limit of detection.

**Table 3:** Evaluation of soil phytotoxicity and PTE concentrations in leaves of *Betula middendorffi* and *Duschekia fruticosa*.

Parameters		Pb	Ni	Mn	Cd	Co	Cr	Zn	Cu	As
Soils										
* F		100.0–400.0	100.0	1500–3000	3.0–8.0	25.0–50.0	75.0–100.0	70.0–250.0	60.0–100.0	15.0–50.0
General for soils of the study area	Mean Max	2.21 6.60	15.39 163.8	311.63 1856.0	0.14 1.12	4.53 34.65	1.94 21.45	12.10 36.46	8.21 28.44	0.22 0.84
	** KF	0.07	**1.64**	**1.24**	0.37	**1.41**	0.29	0.52	0.47	0.06
Soils in the impact zone of the Udachny quarry		1.09	2.89	167.7	0.123	2.79	0.48	14.59	10.54	0.03
KF1		0.01	0.02	0.11	0.04	0.11	0.01	0.21	0.17	0.002
Soils in the impact zone of waste rock dumps	Udachny quarry	2.39	148.5	1770.6	0.059	34.65	16.44	13.83	18.01	0.41
	KF2	0.02	**1.48**	**1.18**	0.02	**1.41**	0.22	0.19	0.3	0.03
	Zarnitsa quarry	4.45	2.64	1856.0	0.440	3.13	0.74	23.85	4.02	0.07
	KF3	0.04	0.03	**1.23**	0.14	0.12	0.04	0.34	0.07	0.004
Tailings pond		1.69	3.07	1463.0	0.134	9.40	0.48	11.59	16.25	0.38
KF4		0.02	0.03	0.97	0.04	0.38	0.06	0.16	0.27	0.02
Plants										
Plants of terrestrial land ***		1.25–1.50	1.54	6.86	0.035–4.40	1.37	1.03	11.76–30.0	2.27–8.0	0.2 ****
*Betula middendorffii*		3.03	8.08	144.8	0.04	0.39	12.30	42.94	4.73	n/d
*Duschekia fruticosa*		3.09	6.81	31.5	0.03	0.15	12.08	n/d	5.16	n/d
Concentration of elements in mature leaf tissues based on generalized data from many species [44]	Deficiency or less than the established required quantities of elements	-	-	15.0–25.0	-	-	-	10.0–20.0	2.0–5.0	-
	Sufficient or normal	5.0–10.0	0.1–5.0	20.0–300.0	0.05–0.2	0.02–1.0	0.1–0.5	27.0–150.0	5.0–30.0	1.0–1.7
	Excess or toxic	30.0–300.0	10.0–100.0	300.0–500.0	5.0–30.0	15.0–50.0	5.0–30.0	100.0–400.0	20.0–100.0	5.0–20.0

Note: * F—total concentrations of microelements in the surface layer of soils, considered to be the limit in terms of phytotoxicity [44]; ** KF—ratio of maximum concentrations to the lower limit of phytotoxicity; ***—intensity of biological absorption of trace elements by terrestrial plants [45,46]; ****—the As content in plants is given according to [47]. Values >1 are marked in bold. By yellow are marked values with deficiency or less than the established required quantities of elements; by green—sufficient or normal values; by brown—excess or toxic values.

**Table 4:** Bioaccumulation factor (*BAF*) in leaves of plants in the study area.

Vegetation Species	Parameters	Pb	Ni	Mn	Cd	Co	Cr	Zn	Cu	As
*Betula middendorffii* (n = 36)	Mean	2.07	3.26	2.32	0.36	0.16	18.85	10.15	1.02	-
	Median	1.39	3.00	0.22	0.28	0.08	13.70	3.22	0.76	-
	Geom. mean	1.62	2.25	0.27	0.29	0.09	12.30	3.77	0.76	-
	Minimum	0.39	0.07	0.02	0.06	0.01	0.52	0.99	0.18	-
	Maximum	9.64	8.40	64.36	1.07	0.78	135.6	210.0	4.27	-
	Std. Dev.	1.76	1.91	10.75	0.25	0.19	22.89	34.60	0.84	-
*Duschekia fruticosa* (n = 14)	Mean	1.86	3.24	0.26	0.25	0.08	15.97	3.43	1.21	-
	Median	2.15	2.73	0.19	0.26	0.06	14.46	2.39	0.88	-
	Geom. mean	1.58	2.68	0.18	0.23	0.07	15.01	2.71	0.95	-
	Minimum	0.65	0.55	0.02	0.06	0.03	8.05	1.05	0.24	-
	Maximum	3.23	8.59	0.97	0.41	0.24	26.66	10.55	2.90	-
	Std. Dev.	0.98	2.05	0.24	0.08	0.06	5.92	2.83	0.81	-

## Data Availability

Data are contained within the article.
